# Evaluating evidence-based health care teaching and learning in the undergraduate human nutrition; occupational therapy; physiotherapy; and speech, language and hearing therapy programs at a sub-Saharan African academic institution

**DOI:** 10.1371/journal.pone.0172199

**Published:** 2017-02-16

**Authors:** Anel Schoonees, Anke Rohwer, Taryn Young

**Affiliations:** Centre for Evidence-based Health Care, Faculty of Medicine and Health Sciences, Stellenbosch University, Cape Town, South Africa; TNO, NETHERLANDS

## Abstract

**Background:**

It is important that all undergraduate healthcare students are equipped with evidence-based health care (EBHC) knowledge and skills to encourage evidence-informed decision-making after graduation. We assessed EBHC teaching and learning in undergraduate human nutrition (HN); occupational therapy (OT); physiotherapy (PT); and speech, language and hearing therapy (SPLH) programs at a sub-Saharan African university.

**Methods:**

We used methodological triangulation to obtain a comprehensive understanding of EBHC teaching and learning: (1) through a document review of module guides, we identified learning outcomes related to pre-specified EBHC competencies; we conducted (2) focus group discussions and interviews of lecturers to obtain their perspectives on EBHC and on EBHC teaching and learning; and we (3) invited final year students (2013) and 2012 graduates to complete an online survey on EBHC attitudes, self-perceived EBHC competence, and their experience of EBHC teaching and learning.

**Results:**

We reviewed all module outlines (n = 89) from HN, PT and SLHT. The OT curriculum was being revised at that time and could not be included. Six lecturers each from HN and OT, and five lecturers each from PT and SLHT participated in the focus groups. Thirty percent (53/176) of invited students responded to the survey. EBHC competencies were addressed to varying degrees in the four programs, although EBHC teaching and learning mostly occurred implicitly. Learning outcomes referring to EBHC focused on enabling competencies (e.g., critical thinking, biostatistics, epidemiology) and were concentrated in theoretical modules. Key competencies (e.g., asking questions, searching databases, critical appraisal) were rarely addressed explicitly. Students felt that EBHC learning should be integrated throughout the four year study period to allow for repetition, consolidation and application of knowledge and skills. Lecturers highlighted several challenges to teaching and practising EBHC, including lack of evidence relevant to the African context and lack of time within curricula.

## Introduction

Evidence-based health care (EBHC) involves integration of best current evidence with the health professional’s expertise and experience and the patient’s values and preferences within the context and feasibility of the local healthcare system [1]. The term EBHC, also commonly known as evidence-based practice, evidence-based decision-making and evidence-based medicine, was first published in 1991 but the concept dates back to the middle of the 20^th^ century [2]. In the 21^st^ century EBHC developed steadily with the ultimate aim to improve patient care by doing more good than harm, to provide a way of dealing with the explosion of scientific literature, avoiding waste of scarce resources, and to allow for public accountability. EBHC gives healthcare professionals a five step framework to use when addressing uncertainty in healthcare decision-making and encourages critical thinking [3–5]. However, EBHC is not only a science but also an art which requires professionals to have insight and develop their own style. EBHC is an evolving field that has been criticised by some, and has occasionally been distorted and misused [6, 7]. Subsequently, the term “real evidence based medicine” has been coined which includes “expert judgement rather than mechanical rule following” and “builds on a strong clinician-patient relationship and the human aspects of care” [7].

The importance of including EBHC in undergraduate health curricula has been widely emphasised [4, 8] with Paul Glasziou pointing out that “the search engine is now as essential as the stethoscope” [9]. The content of EBHC curricula should be based on the five steps of EBHC, namely formulating a specific question, searching for and identifying relevant research to answer the question, critically appraising and interpreting the research, considering the application of research findings, and evaluating and monitoring the healthcare decision and outcomes [4]. These must be built on a foundation of enabling competencies that include biostatistics, epidemiology, basic searching skills and critical thinking [10].

EBHC should ideally be integrated in the clinical setting in order to facilitate bedside teaching and learning, as it has shown to be more effective and relevant to the students than a stand-alone EBHC module [11].

Although EBHC originated in the medical field, it is applicable and relevant to all healthcare professions including the allied health field [4, 8, 12]. According to the Association of Schools of Allied Health Professions (ASAHP), allied health professionals comprise almost 60% of healthcare workers and are those that “deliver services involving the identification, evaluation and prevention of disease and disorders; dietary and nutrition services, and rehabilitation and health systems management” [13]. They support doctors and nurses by functioning in a multi-disciplinary team. There are some published studies showing benefits of including EBHC teaching in undergraduate allied health curricula [14–17], but compared to the medical field, such evidence is sparse [12]. Nonetheless, incorporating the principles of EBHC in the decision-making process is equally important across all healthcare professions to ensure best patient outcomes [4, 12].

In order to develop, extend and strengthen EBHC training in undergraduate programs in South Africa and Africa, the Centre for Evidence-based Health Care (CEBHC) is promoting the integration of EBHC in undergraduate curricula of all healthcare professionals. Our work involves conducting situational analyses of current EBHC teaching and learning across all healthcare disciplines, developing and piloting a model of integrated teaching of EBHC, as well as teaching and assessing EBHC competencies. Previously, the CEBHC conducted a baseline evaluation of EBHC teaching and learning in the undergraduate MB,ChB (bachelor degrees of medicine and surgery) curriculum of a South African university [18, 19]. In this paper we report on the findings of a similar situational analysis of EBHC teaching and learning in the undergraduate allied health programs at a sub-Saharan African academic institution.

Our overarching goal was to identify gaps regarding EBHC competencies in the current undergraduate allied health programs and to suggest where and how EBHC learning can be further incorporated. This was achieved through the following objectives: to (1) assess what EBHC competencies were addressed in the curricula of the undergraduate human nutrition (HN); occupational therapy (OT); physiotherapy (PT); and speech, language and hearing therapy (SLHT) programs; to (2) explore lecturers’ views and experiences of EBHC and of EBHC teaching and learning; to (3) assess students’ self-perceived confidence to practice EBHC, their attitudes towards EBHC and their perceptions about EBHC teaching and learning in their undergraduate programs; and to (4) identify opportunities to optimise integrated EBHC teaching and learning in these programs.

## Methods

A task team consisting of representatives of all four allied health divisions of the specific academic institution and CEBHC researchers was formed to guide the project. The overarching goal and methodology of the project was discussed and agreed upon where after the CEBHC conducted the study, and engaged with the team to report on the progress of the study. In our paper, the term ‘teaching’ refers to the act of a lecturer conveying certain knowledge, skills, attitude, or more than one of these; whereas the term ‘learning’ refers to what the students acquired in terms of knowledge, skills, attitude or more than one of these. Lecturers can have perceptions about the teaching they have done (taught curricula), as well as on the learning process and success of the students; and similarly students can have perceptions about the teaching that they have received, and the learning that occurred (learned curricula) [20].

Each of the four allied health programs is a four year degree program. Typically, students who register for these courses come straight from secondary school (i.e. students typically are 19 years old in first year, and 22 years old when they graduate).

Our evaluation comprised three steps namely (1) a document review of the curricula; (2) focus groups or interviews with the lecturers involved in the undergraduate HN, OT, PT and SLHT programs; and (3) a survey with students. We obtained ethics approval from the Stellenbosch University Health Research Ethics Committee (S12/10/261).

### Document review

Detailed methods of the document review have been previously published [21]. We obtained and reviewed all the relevant documents related to the curricula (as it was in 2012) of the undergraduate HN, PT and SLHT programs, including study guides and module outlines. The OT curriculum was being revised at the time of our study and we could therefore not include it in our document review. We identified learning outcomes related to the set of enabling and key EBHC competencies previously developed and published [18] and also accepted by the institution that we evaluated, as reference standard. One author (AS) developed codes related to the EBHC competencies and compiled a code book, which was piloted and refined accordingly. A research assistant (MZ) read through all the module guides and coded those learning outcomes that related to the pre-specified key and enabling EBHC competencies, using Atlas.ti software. A second author (AR) checked the coding, where after AR and AS discussed all the coded learning outcomes and collated the results in tables, one for each of the programs. Each table contains the set of key and enabling EBHC competencies, a short description of the content addressed, the year of study and module within which competencies were present, the type of learning outcome (according to Bloom’s taxonomy [22, 23]), and an example of such a learning outcome from one of the modules. The tables are a form of an evidence map, where EBHC competencies that were not addressed in any of the modules, were also listed to clearly indicate where the gaps are.

### Interviews with lecturers involved in the four allied health undergraduate programs

We interviewed lecturers across all four undergraduate programs using focus group discussions. We contacted the head of each of the four divisions to suggest lecturers (junior and senior) that we could invite to participate in the focus groups. We invited 7 lecturers from SLHT, 6 each from HN and OT, and 5 from PT. We invited lecturers via email and explained the purpose of the study and focus group discussions, that participation was voluntary and that anonymity was ensured. Lecturers agreed to participate via email. An independent, experienced interviewer with expertise in qualitative research facilitated the focus group discussions and interviews. Separate focus groups were held for each of the four allied health fields. Where key lecturers could not attend the scheduled focus group discussion, we arranged individual interviews. Lecturers’ perspectives on EBHC and the teaching and learning of EBHC in the relevant programs were explored with a semi-structured discussion schedule (Box 1).

**Box 1. Semi-structured discussion schedule for the focus groups and interviews with key lecturers**

What is your understanding of EBHC? What do you think is the importance/value of EBHC? What do you think is the potential challenges of practicing EBHC? How important do you think is it that undergraduate students have EBHC competencies when they graduate? Describe the current method of teaching EBHC in your program. What will help to make the teaching of EBHC easier? Describe the current method of assessing students’ EBHC competencies. What would you say is your students’ attitude and confidence towards practicing EBHC?

The facilitator recorded all discussions with a digital voice recorder (with the verbal consent of the interviewees) and transcribed recordings for analysis purposes. Transcripts were coded using Atlas.ti software (version 7) and extracted data was analysed. One author coded all data (AR), where after all codes were discussed with a second author (AS). We resolved discrepancies by discussion and reached consensus. Qualitative analyses were performed by identifying emerging themes. We reported all relevant data as anonymised quotes to support the results.

### Survey of students

We invited 2013 final year students, as well as 2012 graduates, of each of the four undergraduate allied health programs to complete an electronic survey. We included an incentive for participating in the survey, whereby students completing the survey were offered the opportunity to be entered into a lucky draw to win Trisha Greenhalgh’s book titled “How to read a paper” [24]. The survey (S1 File) explored students’ attitudes towards EBHC, self-perceived competence in EBHC as well as their experience of EBHC teaching and learning during their degree program. The survey was designed using Google forms and a research assistant (OK) piloted it for quality purposes. After finalisation, we sent the link to the survey to students via email. The survey consisted of questions requiring students to rate the appropriateness of the EBHC curriculum and their attitude towards EBHC using a Likert scale [25], as well as questions to determine self-perceived confidence in EBHC using a confidence scale of 10% to 100% [26]. In addition, open-ended questions designed to glean students’ perceptions of EBHC learning during their undergraduate program were included. Participation in the survey was voluntary and anonymity was assured, while completion of the survey was regarded as informed consent. Students were asked to provide us with a mobile phone number if they wanted to participate in the lucky draw; which was used for the sole purpose of notifying the winning participant.

Quantitative results were analysed descriptively with the help of Stata software (version 12). Qualitative data from open-ended questions was coded with Atlas.ti software (version 7) by one author (AS) and discussed with a second author (AR). Qualitative analyses were performed by identifying emerging themes.

We triangulated results of the document review, focus groups with lecturers and survey with students by identifying cross-cutting themes and presenting them in a narrative synthesis. For each theme, we reported on the similarities and differences (if applicable) across the three study steps.

After completion of all three components of this study, an informal dissemination session with the task team and other interested lecturers from the four allied health divisions was held. We shared and discussed our methodology and all relevant data (descriptive, quantitative and qualitative) from the document review, focus groups with lecturers, and student survey.

## Results

### Description of the included data

#### Document review

We reviewed a total of 89 module guides, 37 from SLHT, 29 from HN and 23 from PT. Of these, HN and PT share four and three modules respectively with the medical (MB,ChB) program; HN, OT and PT share one module; OT and PT share two modules; OT and SLHT share five modules; and OT, PT and SLHT share one module over the four study years.

#### Interviews with lecturers

Independent researchers facilitated four focus group discussions, one for each of the four programs. They also conducted two interviews with key lecturers that were unable to attend the focus groups. Six lecturers each from HN and OT, and five lecturers each from PT and SLHT participated.

#### Survey respondents

We were unable to obtain the email addresses of all 2012 graduates from the various undergraduate programs. We thus invited the 2012 graduates which we could contact (16 OT graduates, 33 PT graduates, and none from HN or SLHT) together with all 2013 final year students across all four programs (26 HN, 35 OT, 42 PT, and 24 SLHT students). Thirty percent (53/176) of invited students responded to the survey, of which two were male and 51 female; and six were 2012 graduates and 47 were final year students of 2013. The breakdown of the respondents is presented in Table 1. Furthermore, the average and median age of the survey respondents was 22.5 years old, with the range between 21 and 35 years. Three of the 53 respondents–all three PT students–had previously completed a different undergraduate degree (BSc Sport Science, BSc Zoology and Biochemistry, and BSc Forestry). For the other 50 respondents the specific allied health degree was their first degree.

**Table 1:** Responses to student survey according to allied health program.

Course	Invited (n)	Respondents (n)	% of total responses
PT	75	34	64
HN	26	9	17
OT	51	8	15
SLHT	24	2	4
Total	176	53	100%

### Informal dissemination session with lecturers

Six lecturers, at least one of each program, attended the informal dissemination and feedback session.

### Description of triangulated findings

We narratively synthesised results according to themes cutting across all three steps of this project. Supporting data include detailed results of the document review in tables (S1 Table), quotes of the qualitative analyses of the focus groups and interviews with lecturers (Table 2), and the findings of the student survey are presented in Tables 3 and 4 as well as in S1 and S2 Figs.

**Table 2:** Selected quotes from focus group discussions and interviews with lecturers.

**EBHC competencies**
“I would think in the first two years… the focus is not that much on evidence, during the first two years the focus is much more on knowledge and insight. Whereas in the third and fourth year the focus moves towards application.”
“One of the things I do in my particular area is right at the beginning of the block I tell them that they got to identify an issue whether it is an evaluation issue, a treatment decision making problem, what am I going to do in this scenario, whatever it is, they have to formulate a question.”
“…I think the students get exposure to that… and I think that is what we don’t sometimes do explicitly, but we give it implicitly to them in a way you give a lecture or how you appraise or you give them a journal article. They must do the discussions in class and I think there are gaps: we do it for the reason so that you can learn to look at it critically and all those aspects. But we don’t necessarily make it explicitly as an outcome at the moment in our lesson materials.”
**Approach to teaching**
“They have a specific task in the first year where we teach them in nutrition, how to look at scientific evidence. So we give them the red flags to look out for in articles and science and then they have to go and source different articles: the journal article, media article etc. and then they have to tell us what are the signs that you look for, that this is not a true science.”
“We actually do an interesting thing in the fourth year too when they basically finished the research project then we have a task engaging the media and we use a well-known professor in journalism too and he then actually take them from searching the evidence and then writing it in the language that the media would interact with.”
“I think the fact that their research requires them to do a systematic review which is one way of really teaching critical thinking and looking for evidence and they have to complete that with the patient.”
**Value of teaching EBHC**
“I still sometimes get emails from students that qualified under the old curriculum before we actually changed to problem based learning… and then they ask me for evidence. One of their patients asked for evidence that this and this was working, can I just send them an article? So the first thing that I think of like they haven’t got a clue where to go and find the evidence so they are definitely not using it in their own practice and so they are basically stuck with what we taught them twenty years ago.”
“Hoping obviously that by the time that they leave, that they know where to source information, how to critically analyse it, how to come to an evidence based recommendation.”
**Assessment of EBHC**
“The formal evaluation would be in research methodology in that specific course in the third year… whereas with all the others it will be interlinked with everything else that we do.”
“I don’t think we focus on evidence based healthcare as a topic when we plan our assessment.”
“So it’s a more indirect assessment of the skill to dissect information.”
**Challenges in teaching EBHC**
“I think for us to develop a skill we need repetition and practice and I think you need time for that and I think that’s something that within our programmes is very difficult to find. So you try to find more than one module or theme or activity in which to expose the students to and for them to get practicing but I think because for it being such a full programme you are not going to get to a place where you can say like have done this, that when they leave at the end of four years that you can say that they are efficient in all these things.”
“So we’ve got a certain amount of prescribed work that you have to put across and textbooks are basically old in majority of cases. I mean certain concepts will forever be the same but new stuff always appears so we use the textbook as the basis because that is the way that we have to teach. And then in class you always have to juggle how much new information do I put forward because, I mean, we tend to probably go the route of information overload when we teach to our students.”
“I know the clinicians out there are not using this approach…”
“The schooling system where they come from has a huge impact. [If] They were used to being challenged and think for themselves and it’s easier for them to adapt but if they have been to a school where they have to memorise it is very difficult for them to accept that challenge.”
**Challenges in practicing EBHC**
“There’s a general consensus in the profession that there is not a lot of evidence for what we do. So people are aware about the lack of evidence.”
“It can’t be directly applied and I think that’s the thing. So even if evidence is done in a beautiful large randomised controlled trial it still is not something if it’s done in North America really. It’s just not going to be useful to a child who speaks Zulu in KwaZulu Natal you know.”
“I think our field is perhaps particularly challenging because of the various angles from which there is so called evidence. So you have got the journal articles but there is the media and the experts and it’s a difficult field to actually get to the truth from a student’s perspective.”
“Well if they find evidence it’s often… not necessarily representative of what you would actually do when you go into a clinical setting. So it would be like very intensive therapy for three hours a week which no one would be able to do except if they are in a really good clinical setup.”
**Attitudes towards EBHC**
“For me…it’s non-negotiable.”
“So there are people who will sort of strongly scribe to it but maybe here in South Africa it is slightly different.”
“And what we must tell you is that there is resistance from the students. They would very much prefer to be given a set of guidelines, to be given a programme because that makes them feel safe and I often encounter that they feel quite unsafe if I tell them, look I don’t know either, let’s start the process of clinical reasoning. That’s not what they want to hear, they want to hear that we know everything.”
**Understanding of EBHC**
“So it’s a whole integration of your expertise, best evidence, the patient, the context.”
“I think it’s basically a lifelong skill that you need to acquire to use in the future… because in twenty years things could change radically so they need to know where to go and find the best evidence and how to use that.”
“Not just being able to know where to go and get it. But to be able to reflect and evaluate and critically analyse that research as well.”
**Misperceptions of EBHC**
“It’s basically that you hope that there is current evidence just to prove that this that we are doing for our patients’ treatments or assessments has been proven in the literature.”
“But I think there’s an area that we haven’t explored yet and that is practise knowledge as best evidence and it is giving too little value in your typical traditional medical setting or medical model. And I think the traditional hierarchy of evidence… excludes practise based knowledge or it would see it almost as…the lowest level.”
“Only certain types of research are acknowledged.”
**Value of EBHC**
“It gives you confidence and also credibility when you have discussions with people who can make a change… when it comes to policy development…But you can’t just come and say but it works or this is what I do and it is effective and we must implement it. We need to have evidence that must be published and it gives you credibility so yes it gives you a whole lot of skill sets but also credibility.”
“Yes, it’s there where the three prongs come in because I think in a South African context that whole contextual thing weighs more than anything else and the patient values and it’s just a completely different ball game.”
**Criticisms of EBHC**
“I think it limits your thinking, it limits collaboration and inter professional collaboration…”
“It’s just not applicable to our profession.”
“I am very against medical based knowledge in the sense that I think it is very technical and it’s not humanistic and I think that… it doesn’t take context into consideration at all and I think… the way that we generate information is based on so many factors. It’s very complex and medical doctors usually don’t understand it at all and maybe sometimes we don’t understand it ourselves because a lot of it is instinctual knowledge.”
“I think a lot of that [EBHC] is primarily promoting a way of thinking about research that means you can understand people, you can objectify them, you can manipulate variables, you can definitely generalise data and all of that. Which is maybe against what other people think…”
“I would not personally like not to see it as a given but as an approach and that should be critically evaluated in itself.”

**Table 3:** Selected quotes from open-ended questions in the student survey.

**EBHC competencies**
“Many students, still, cannot read a paper correctly.”
“I don't think the use of EBP was pushed well enough. I feel that many of the students still just plod along doing the basics they learned in class and are not challenging themselves. I also see students using methods there is no evidence for and not even indicated for the condition they are treating. I think the students "box" their techniques because they are not encouraged enough to challenge the norms. Also there is not enough repetition of techniques throughout the 4 years of study, i.e. we learn a technique, then it may be mentioned in passing once again during the course.”
“It was very thoroughly done and notes and lectures were continuously updated according to how literature changed.”
“Sometimes feel we [are] in charge of our own notes and to attain our own knowledge.”
**Approach to teaching**
“I think we should be trained more on how to look for evidence based information.”
“Not to ‘spoon feed’ us, but to close the gap between spending hours looking for a good article and finding the information you are looking for.”
“Would have liked more about practical application and how to read an article.”
“Extended classes on different types of studies and how to identify them.” (translated from Afrikaans)
“Teaching of evidence-based health care was sufficient, but too little emphasis was placed on how to go to work to do it yourself.”
“I found it difficult to apply the theory learned in lectures with actual experience or real-life examples.”
**Challenges in teaching EBHC**
“There was not enough time allocated to critically examine and discuss different literature to actively learn in the lecture environment.”
“…more emphasis [to be] placed on already implementing evidence-based health care during our clinical rotations. Then, we would have really realised the importance of this in different clinical situations, and experienced the practical side of it, rather than just studying it as theoretical knowledge and having some practice in it.”
**Attitudes towards EBHC**
“It is very important to our lecturers that we find out for ourselves and find the evidence for it rather than just accepting what they say.”
“It makes a student more independent and creates a culture of continuous learning.” (translated from Afrikaans)
**Misperceptions of EBHC**
“I believe most and all our work are based on evidence-based health. We have a lot of extra articles to read.”
“Backing up our proposed treatments with evidence”
“We were encouraged to make use of only based research during evidence our studies.”
**Improving EBHC teaching**
“…more emphasis [to be] placed on already implementing evidence-based health care during our clinical rotations. Then, we would have really realised the importance of this in different clinical situations, and experienced the practical side of it, rather than just studying it as theoretical knowledge and having some practice in it.”
“More attention needs to be placed on using literature in conjunction with the notes by emphasising the importance of literature…”
“Cases are good and you develop very good clinical reasoning skills, but there is gaps in our knowledge. Except for cases, we have nothing to fall back on, especially in a field like ICU PT. So most of our class is using notes and of other universities to help us. It would be really nice if we can also get the information.”
“There was not enough time allocated to critically examine and discuss different literature to actively learn in the lecture environment.”
“Encourage it from earlier on in the course (1st year) so that it becomes a trend and a habit.”
“I think we need to be taught research principles and how to do referencing and search for information from first year (more in-depth). . . to allow for more time… to process work… and… repetition throughout the years.”

**Table 4:** Survey results of students on coverage of evidence-based health care competencies within allied health curricula.

To what extent were the following topics on EBHC covered in the allied health program? Total n = 53	Not at all n (%)	Inadequate n (%)	Basic n (%)	Adequate n (%)	Comprehensive n (%)
Identifying a personal gap in knowledge.	0	8 (15)	17 (33)	23 (43)	5 (9)
Formulating an answerable research question using the PICO process.	0	4 (8)	13 (25)	22 (41)	14 (26)
Developing a search strategy based on the PICO question.	0	5 (9)	17 (32)	18 (34)	13 (25)
Doing a thorough literature search related to a question you have.	0	2 (4)	8 (15)	28 (53)	15 (28)
Distinguishing between different types of studies.	0	0	8 (15)	27 (51)	18 (34)
Identifying study designs relevant to a question.	0	4 (8)	20 (38)	17 (32)	12 (23)
Critically appraising the quality of different study designs.	1 (2)	5 (9)	11 (21)	21 (40)	15 (28)
Interpreting the results of studies.	0	4 (8)	18 (34)	26 (49)	5 (9)
Applying the findings to your clinical setting by considering the evidence, your own clinical experience and individual patients.	1 (2)	2 (4)	19 (36)	23 (43)	8 (15)
Evaluating the process of EBHC on an on-going basis.	0	5 (9)	23 (43)	16 (30)	9 (17)

#### Evidence-based health care competencies in the allied health curricula

Results from the document review showed that EBHC learning outcomes were concentrated in specific theoretical modules (e.g. years 3 and 4 research methods modules) and were generally not addressed in clinical modules (see S1 Table for detailed findings). This finding was confirmed by the lecturers who said that EBHC key competencies were mostly taught in the third and fourth year of the four year degree programs. However, these competencies were mostly covert and addressed as a whole and not as separate, explicit steps of the EBHC process. One aspect which stood out was that, although the key competency critical appraisal was generally addressed (e.g. “develop critical skills in evaluating research”), none of the learning outcomes referred to critical appraisal of specific study designs (e.g. RCT’s, cohort studies, etc.). Enabling competencies such as epidemiology and biostatistics were addressed in specific theoretical modules (e.g. “Health in context” in first year, “Research methodology” in third year), while critical thinking, communication and reflective practices were emphasised throughout the programs. Most respondents to the survey felt that EBHC competencies were covered to a basic or adequate extent, while some also thought they were covered comprehensively (Table 4). Although students’ confidence in their EBHC skills varied, the majority was confident that they were able to perform the steps of EBHC (S1 Fig).

#### Approach to teaching evidence-based health care

Lecturers discussed various approaches to teaching EBHC. Among these were some generic techniques like problem-based learning and journal clubs, but also program-specific techniques. The physiotherapy students, for example, needed to conduct a systematic review in their fourth year, taking them through the first three steps of EBHC. Students responding to the survey agreed that this was a good approach to learning the principles of EBHC, since it allowed them to apply the theoretical concepts. Another example of a specific EBHC task in the human nutrition program, required students to look at different sources of evidence (including media articles and scientific evidence) and critically evaluate the information they contained. In their fourth year they also practised how to communicate evidence to the media in plain language. Searching skills across programs were mainly taught to students by means of a hands-on workshop, facilitated by the university’s health sciences library.

Lecturers also stressed the importance of integrating theoretical principals in the clinical setting when making healthcare decisions about patients. Students felt that they wanted more discussion of the literature rather than just being taught the results or conclusions of a study. On the one hand they felt that the theoretical concepts needed to be broken down into basic steps, while on the other hand some felt that EBHC teaching mainly focused on theory and required more application. Results from the document review also showed that EBHC learning outcomes were mainly addressed in theoretical and not in clinical modules.

#### Assessment of evidence-based health care competencies

We were unable to extract information on assessment of students’ EBHC competencies from the documents that we reviewed. Lecturers described assessment of EBHC competencies as being integrated into class discussions and assignments, but not being overt in formal assessments except when relating to modules like Biostatistics and Research methodology, where biostatistics and epidemiological concepts were assessed. Students felt that EBHC needed to get a wider coverage in tests and exams, since they could easily pass with minimal understanding of the concepts. This notion that assessment drives learning (and vice versa) was highlighted by lecturers.

#### Attitudes to evidence-based health care

Lecturers’ attitudes towards the importance of practising EBHC as allied health professionals varied. Some thought of it as being non-negotiable, while others found the concept to be of limited value, especially in an African context.

“For me…it’s non-negotiable.”

“So there are people who will sort of strongly scribe to it but maybe here in South Africa it is slightly different.”

They felt that most students gradually developed a positive attitude towards EBHC during the fourth study year, although some resistance towards it had also been experienced. Students perceived lecturers’ attitude towards EBHC to be positive and results from the survey showed that students themselves also had a positive attitude (S2 Fig).

“It is very important to our lecturers that we find out for ourselves and find the evidence for it rather than just accepting what they say.”

#### Challenges related to teaching and practicing evidence-based health care

Challenges that lecturers experienced when teaching EBHC included lack of time in the program to dedicate to EBHC, lack of role models in the clinical field, lack of student motivation, lack of evidence related to a specific field and related to the African context, schooling background, language difficulties and assessment of EBHC skills.

“I think for us to develop a skill we need repetition and practice and I think you need time for that and I think that’s something that within our programmes is very difficult to find. So you try to find more than one module or theme or activity in which to expose the students to and for them to get practising but I think because for it being such a full programme you are not going to get to a place where you can say like have done this, that when they leave at the end of four years that you can say that they are efficient in all these things.”

Students echoed some of these challenges, and especially highlighted the lack of time and opportunity to apply their theoretical knowledge of EBHC in the clinical field. They felt that they lacked skills and confidence to efficiently find and appraise the evidence.

“…more emphasis [needs to be] placed on already implementing evidence-based health care during our clinical rotations. Then, we would have really realised the importance of this in different clinical situations, and experienced the practical side of it, rather than just studying it as theoretical knowledge and having some practice in it”.

Lecturers also referred to a number of challenges when practicing EBHC on a daily basis. These included the lack of evidence, the sources of evidence, the applicability of evidence, lack of access to evidence, lack of EBHC knowledge and skills, lack of time and information overload.

“It can’t be directly applied and I think that’s the thing. So even if evidence is done in a beautiful large randomised controlled trial it still is not something if it’s done in North America really. It’s just not going to be useful to a child who speaks Zulu in KwaZulu Natal you know”. (SLHT lecturer)

#### Perceptions about evidence-based health care

Understanding of EBHC as a concept differed between lecturers. Some described it according to the three principles of EBHC, namely integrating current best evidence, clinical expertise and patient values; while others perceived it as a lifelong learning skill that all healthcare professionals should have in order to stay up to date. Lecturers also stressed that EBHC should focus on the application of evidence in daily practice and should not remain a theoretical skill.

“I think it’s basically a lifelong skill that you need to acquire to use in the future… because in twenty years things could change radically so they need to know where to go and find the best evidence and how to use that.”

Misperceptions about EBHC and its application also emerged. Amongst these misperceptions was the notion that EBHC only considered randomised controlled trials and that other study designs had no value, which renders it unsuitable for certain healthcare professions. Another view was that clinical expertise was ignored in an evidence-informed approach. Others felt that they needed peer-reviewed evidence as proof that their current practices were valid, which relates to cherry picking of results. Some lecturers said that they made sure the content they were teaching was based on evidence and that they made sure to include a lot of references to published articles when teaching. Some students were under the impression that referring to *any* scientific, peer-reviewed article as opposed to websites and media articles constituted EBHC.

“Only certain types of research are acknowledged.”

“It’s basically that you hope that there is current evidence just to prove that this that we are doing for our patients’ treatments or assessments has been proven in the literature.”

Some lecturers criticised EBHC and felt that it limited one’s thinking by having a narrow approach to a problem; that it did not take the context into consideration and that it was one dimensional and too medically orientated.

“I think it limits your thinking, it limits collaboration and inter professional collaboration…”

“It’s just not applicable to our profession.”

Lecturers also discussed the value of EBHC and described it as improving patient care, rendering credibility to one’s practices and enabling inclusion of ethical considerations in a healthcare decision. They highlighted the importance of considering the African context when practicing in an evidence-informed manner. Students felt that EBHC was important because it taught them to be accountable for their healthcare decisions and their patients.

“It gives you confidence and also credibility when you have discussions with people who can make a change… when it comes to policy development…But you can’t just come and say but it works or this is what I do and it is effective and we must implement it. We need to have evidence that must be published and it gives you credibility so yes it gives you a whole lot of skill sets but also credibility.”

#### Improving evidence-based health care teaching

Students responding to the survey suggested that study notes should contain both notes from the lecturer, as well as information from the literature in an integrated manner. Although they felt that the theoretical concepts of EBHC were well presented, they highlighted the need for consolidation of concepts and application of theoretical concepts in the clinical field. They wanted to feel competent in making independent decisions. They also felt confused by the different sources of evidence (e.g. internet, textbook, notes, scientific literature, lecturers), which sometimes gave opposing information and felt that they lacked critical appraisal skills. Students recommended that EBHC should be taught from first year, so that it can be repeated during the course of the four years and can become a habit.

“Encourage it from earlier on in the course (1st year) so that it becomes a trend and a habit.”

“More contact time in terms of evidence based teaching in discussion form rather than ‘reading from slides’ or just discussing the content of the slides (which is often in a prescribed textbook).”

Lecturers, however, felt that certain EBHC competencies such as formal critical appraisal of articles should be taught at postgraduate or even continued education level and not at undergraduate level.

## Discussion

Results from triangulation of methods show that EBHC competencies were partly addressed in the four allied health curricula, although these consisted mainly of enabling competencies. There was a lack of integrated teaching of EBHC in the clinical modules and some students felt that they were not adequately equipped to make independent healthcare decisions, informed by the current best evidence. This finding is consistent with other studies in the literature, where allied health students acknowledge the importance of EBHC, but do not practise it themselves [27–33]. For example, Gray et al. [31] assessed 231 occupational therapists in their first year of practice from Australia and New Zealand with an online survey and found that practising EBHC was among the lowest ranked competencies. The importance of integrating EBHC teaching into clinical lectures has been highlighted by other studies in the allied health field [27, 28, 30, 33, 34]. In our study, lecturers felt that EBHC was being taught sufficiently, although this mostly happened in an implicit manner and was not explicitly reflected in learning outcomes. With *explicit* learning the specific knowledge or skill is taught deliberately and the learning outcome is known to students, while *implicit* learning refers to incidental learning or hidden learning [35]. Boruff and Thomas [27] stated that if OT and PT professionals are to “successfully apply literature searching and critical appraisal in clinical practice, they must acquire these skills early in their career and ideally during their formal training”. Furthermore, Spek et al. [32], suggested that developers of EBHC (undergraduate) curricula should give “explicit attention” on improving the practising of EBHC.

We found that most lecturers had a positive attitude towards EBHC, while some felt that it was not applicable to the allied health professions. This was partly based on an underlying misperception that EBHC considers results from randomised controlled trials (RCTs) only and does not value other study designs which are more common in the allied health fields. This perception has also been identified as a barrier to practicing EBHC in the allied health field [12]. While RCTs are seen as the best study design to answer questions related to interventions and treatments, questions about risk factors, harms, diagnosis and patient experiences require other study designs, such as illustrated by the Centre for Evidence-based Medicine in Oxford (www.cebm.net) hierarchy of evidence, based on the different types of questions [36].

In addition, lecturers raised the issue of timing of EBHC teaching during the dissemination meeting as they did not feel that all the EBHC competencies should be taught in the undergraduate programs, mostly due to the full clinical and community curricula. Students on the other hand, disagreed with lecturers and proposed that EBHC teaching should begin in the first year of study, leaving enough time for repetition, consolidation and application of knowledge and skills. International teachers of EBHC also advocate that the foundation of EBHC principles should be firmly laid at undergraduate level and built on at postgraduate level [4, 7, 37, 38]. If the principles of EBHC are introduced in the early years of study, students will be more likely to incorporate EBHC in their personal way of clinical and community decision making, than when they learn about EBHC after they already have found their own style of making healthcare decisions. Students also highlighted the need for integrated teaching of EBHC which resonates with the international literature on EBHC teaching. Based on findings from a systematic review, Khan and Coomarasamy [39] have produced a hierarchy of EBHC teaching where interactive and clinically integrated teaching of EBHC is viewed as the best method to teach EBHC. An overview of systematic reviews that looked at the effectiveness of EBHC teaching also concluded that multi-faceted, clinically integrated teaching that includes assessment was most likely to increase EBHC knowledge and skills [11].

Lecturers were particularly concerned about the lack of evidence relevant to the South African context and felt that this was a challenge when teaching and practicing EBHC in a culturally diverse environment. Although the lack of evidence generated in Africa is well known and a documented challenge [12, 40, 41], it should not be seen as a hindrance to teaching students EBHC competencies. In addition, the evidence should not be the only factor taken into consideration when making healthcare decisions. Patient values, clinical experience, context, availability and cost of treatment are only some of the other factors that play a role.

EBHC competencies refer to the knowledge and skills needed to formulate clear questions, find the appropriate literature, critically appraise the literature, apply the findings of the evidence and evaluate the process. Teaching students relevant content which is based on the latest evidence does not constitute EBHC teaching. We felt that this caused confusion amongst some of the lecturers on the aim of our study.

It emerged that some lecturers had misperceptions about EBHC and its application, which may influence students’ understanding of the concept. Among these was the idea that EBHC values RCTs only, that evidence supersedes clinical expertise, and that using any published, peer-reviewed article, as opposed to best current evidence, to substantiate one’s practice constitutes “evidence-based” practice. These misperceptions are in line with previous findings [6, 7]. Niemivirta [42] said that increasing knowledge and skills in EBHC only leads to behaviour change when accompanied by the belief that it is desirable and achievable. Lectures should be role models of EBHC [29, 32, 34], to show students that it is attainable, and how. Therefore it is important to debunk myths about EBHC, and to train the trainers in practising and teaching EBHC. Bozzolan et al. [34] investigated the clinical behaviour of 73 undergraduate PT students in Italy, and concluded that the integration of EBHC for students during their internship (final year) “seems to be hampered by the absence of a direct example by the clinical educators”. In Rindflesch et al. [43], the authors interviewed nine third year PT students and explored the characteristics that students want lecturers to have. Being a good teacher (e.g. the ability to receive and give constructive feedback), having insight in guiding students appropriately according to individual needs, and “using evidence-based practice” were the three themes that emerged. Students want lecturers to help them learn how to “integrate current research into clinical practice”. Furthermore, it is important that lecturers as well as professionals working in the clinical field should be trained to teach EBHC and should be role models for practising EBHC. Cameron et al. [29] assessed the utilisation of EBHC among registered OTs in the United States of America, and found that the longer a therapist has been in clinical practice, the more likely will clinical decisions be based on experience rather than evidence. Therefore, professionals in the field, and not just lecturers, are “an ideal target audience” for the training of EBHC [29]. Spek et al. [32] agrees by concluding that “a professional culture in which professionals are competent and confident EBP [evidence-based practice] users would have a positive effect on EBP self-efficacy [student’s belief of how achievable a certain task is] in students”.

### Limitations

Although respondents to the survey made judgements about their self-perceived confidence in practicing EBHC, we did not objectively evaluate students’ EBHC knowledge and skills. In addition, we had a poor response rate to the survey and thus only captured perceptions from a selected group of students. While some views might have been omitted, some students might also feel differently once they start working in the clinical field. Only 11% (6/53) of respondents were 2012 graduates who already had some experience as an independent practitioner.

The research team did not facilitate the focus groups and interviews, because we wanted to give the lecturers an opportunity to voice their true views about EBHC in their curricula, without being influenced by proponents of EBHC. However, when reading the transcripts we came across a number of instances where we would have encouraged participants to elaborate on important issues that they touched on. Although we feel that some potentially useful information was missed, we recognise both the pros and cons of having objective interviewers conduct the focus group discussions.

While there are advantages of grouping the four allied health programs (HN, OT, PT, and SLHT) together for the purpose of a study like ours (e.g. to avoid comparisons between divisions that potentially could imply that one division’s EBHC teaching is better than another; to enhance multidisciplinary corporation; feasibility and best use of resources), there might also be disadvantages to generalise findings across the programs. We acknowledge that there are differences between programs and attitudes towards EBHC amongst lecturers and that some tend to incorporate more EBHC teaching than others; and it is also possible that students from certain allied health programs can be better equipped to practice EBHC when compared to others. This can however differ from university to university. Furthermore, we evaluated one university and thus the findings and experiences cannot necessarily be generalised to every other university.

## Conclusion and recommendations

It is important that all healthcare professionals are equipped with EBHC competencies when they graduate, so that they are able to deal with uncertainty in healthcare decision-making, the information overload they face in their daily practice, and to make more informed decisions about health care for best patient outcomes.

We suggest the following to optimise EBHC teaching in allied health programs:

- include explicit learning outcomes related to EBHC in the module guides;
- introduce EBHC in the first year of the programs (can be a separate module or an explicit component of a module) and scaffold EBHC teaching in subsequent years;
- integrate EBHC teaching in the clinical modules for bedside EBHC learning;
- include EBHC in assessments and provide feedback on these;
- evaluate EBHC curricula on a regular basis;
- organise faculty-wide workshops on curriculum development as well as teaching EBHC in the classroom and at the bedside;
- and collaborate and engage in open discussions with all relevant stakeholders.

## Supporting information

S1 Fig Graphs depicting students’ self-perceived confidence in practicing evidence-based health care. (DOCX)

S2 Fig Graphs depicting students’ attitudes towards practicing evidence-based health care. (DOCX)

S1 File Online survey to students. (DOCX)

S1 Table Results from the document review for three undergraduate allied health programmes. (DOCX)
