# Supplementary material for: Evaluating evidence-based health care teaching and learning in the undergraduate human nutrition; occupational therapy; physiotherapy; and speech, language and hearing therapy programs at a sub-Saharan African academic institution
Source: PLoS One. 2017 Feb 16;12(2):e0172199. doi: 10.1371/journal.pone.0172199 (PMC5313131; doi:10.1371/journal.pone.0172199)
Supplement: S2 Fig — (DOCX) [file pone.0172199.s002.docx]

**S2 Fig. Graphs depicting students’ attitudes towards practicing evidence-based health care.**
