# Supplementary material for: Evaluating evidence-based health care teaching and learning in the undergraduate human nutrition; occupational therapy; physiotherapy; and speech, language and hearing therapy programs at a sub-Saharan African academic institution
Source: PLoS One. 2017 Feb 16;12(2):e0172199. doi: 10.1371/journal.pone.0172199 (PMC5313131; doi:10.1371/journal.pone.0172199)
Supplement: S1 File — (DOCX) [file pone.0172199.s003.docx]

**S1 File. Online survey to students.**

**Demographic data**

*Please complete the following questions before starting the survey:*

**Age:**

**Gender: Male/Female**

**Please indicate which programme you are completing:**

- **Occupational therapy**
- **Physiotherapy**
- **Speech, language and hearing therapy**
- **Nutrition**

**Is this your first degree?**

- **Yes**
- **No**

**If not, please indicate your previous tertiary qualification(s):**

**Cell phone number (if you want to enter the lucky draw):**

| EBHC  Within the four years of your studies, to what extent were the following topics on EBHC **covered** in the curriculum? | | | | | |
| --- | --- | --- | --- | --- | --- |
| Identifying a personal gap in knowledge. | not at all | inadequate | basic | adequate | comprehensive |
| Formulating an answerable research question using the PICO process. | not at all | inadequate | basic | adequate | comprehensive |
| Developing a search strategy based on the PICO question. | not at all | inadequate | basic | adequate | comprehensive |
| Doing a thorough literature search related to a question you have. | not at all | inadequate | basic | adequate | comprehensive |
| Distinguishing between different types of study designs. | not at all | inadequate | basic | adequate | comprehensive |
| Identifying study designs relevant to a specific question. | not at all | inadequate | basic | adequate | comprehensive |
| Critically appraising the quality of different study designs. | not at all | inadequate | basic | adequate | comprehensive |
| Interpreting the results of studies. | not at all | inadequate | basic | adequate | comprehensive |
| Applying the findings to your clinical setting by considering the evidence, your own clinical experience and individual patient’s preferences. | not at all | inadequate | basic | adequate | comprehensive |
| Evaluating your EBHC practice on an ongoing basis. | not at all | inadequate | basic | adequate | comprehensive |

**How did you experience EBHC teaching during your undergraduate studies?**

**Do you have any suggestions how to improve EBHC teaching at undergraduate level?**

**Self-perceived competence (adapted from the EPIC scale (**[**Salbach and Jaglal, 2011**](#_ENREF_18)**)**

***0% =no confidence; 100% = completely confident***

***Please rate your confidence in your ability to:***

- 1. Identify a gap in your knowledge related to a clinical situation

0% 10% 20% 30% 40% 50% 60% 70% 80% 90% 100%

- 1. Formulate answerable questions based on your gap in knowledge

0% 10% 20% 30% 40% 50% 60% 70% 80% 90% 100%

- 1. Develop a search strategy based on the question

0% 10% 20% 30% 40% 50% 60% 70% 80% 90% 100%

- 1. Perform literature searches to address the question

0% 10% 20% 30% 40% 50% 60% 70% 80% 90% 100%

- 1. Use MeSH terms in your search

0% 10% 20% 30% 40% 50% 60% 70% 80% 90% 100%

- 1. Use filters (e.g. sex, date, age) in your search

0% 10% 20% 30% 40% 50% 60% 70% 80% 90% 100%

- 1. Distinguish between different study designs

0% 10% 20% 30% 40% 50% 60% 70% 80% 90% 100%

- 1. Critically appraise the strengths and weaknesses of different study designs

0% 10% 20% 30% 40% 50% 60% 70% 80% 90% 100%

- 1. Select the most relevant study for your question

0% 10% 20% 30% 40% 50% 60% 70% 80% 90% 100%

- 1. Determine the validity of a study based on the methodology

0% 10% 20% 30% 40% 50% 60% 70% 80% 90% 100%

- 1. Interpret the results of a study with ease

0% 10% 20% 30% 40% 50% 60% 70% 80% 90% 100%

- 1. Determine if the evidence obtained is applicable to your clinical setting

0% 10% 20% 30% 40% 50% 60% 70% 80% 90% 100%

- 1. Consider individual patient needs and values

0% 10% 20% 30% 40% 50% 60% 70% 80% 90% 100%

- 1. Decide on an appropriate course of action based on integrating the research evidence, clinical judgement and patient preferences

0% 10% 20% 30% 40% 50% 60% 70% 80% 90% 100%

- 1. Evaluate your EBM practice

0% 10% 20% 30% 40% 50% 60% 70% 80% 90% 100%

**Attitude - adapted from (**[**Baum, 2003**](#_ENREF_1)**)**

**Please rate the following:**

***Scale: 1=strongly disagree to 5=strongly agree***

- 1. EBHC is realistic to practice in routine patient care.

1: strongly disagree; 2: disagree; 3: don’t know; 4: agree; 5: strongly agree

- 1. EBHC is useful on a daily basis.

1: strongly disagree; 2: disagree; 3: don’t know; 4: agree; 5: strongly agree

- 1. I think it is important to practice EBHC on a regular basis.

1: strongly disagree; 2: disagree; 3: don’t know; 4: agree; 5: strongly agree

- 1. I rarely formulate questions about patients.

1: strongly disagree; 2: disagree; 3: don’t know; 4: agree; 5: strongly agree

- 1. Literature searches are too time-consuming to do in a clinic.

1: strongly disagree; 2: disagree; 3: don’t know; 4: agree; 5: strongly agree

- 1. My questions can be answered faster when referring to a textbook or a consultant, than performing the steps of EBHC.

1: strongly disagree; 2: disagree; 3: don’t know; 4: agree; 5: strongly agree

- 1. All types of studies are of equal value to me.

1: strongly disagree; 2: disagree; 3: don’t know; 4: agree; 5: strongly agree

- 1. EBHC is irrelevant to my practice.

1: strongly disagree; 2: disagree; 3: don’t know; 4: agree; 5: strongly agree

- 1. I think EBHC is cookbook medicine where one follows a recipe.

1: strongly disagree; 2: disagree; 3: don’t know; 4: agree; 5: strongly agree

- 1. As a healthcare practitioner, life-long learning is vital.

1: strongly disagree; 2: disagree; 3: don’t know; 4: agree; 5: strongly agree
