# Supplementary material for: Evaluating evidence-based health care teaching and learning in the undergraduate human nutrition; occupational therapy; physiotherapy; and speech, language and hearing therapy programs at a sub-Saharan African academic institution
Source: PLoS One. 2017 Feb 16;12(2):e0172199. doi: 10.1371/journal.pone.0172199 (PMC5313131; doi:10.1371/journal.pone.0172199)
Supplement: S1 Table — (DOCX) [file pone.0172199.s004.docx]

**S1 Table. Results from the document review for three undergraduate allied health programmes.**

**Evidence-based health care competencies per the undergraduate human nutrition programme**

| **Competencies** | **Content covered** | **Year** | **Module** | **Type of learning outcome** | **Quotes of typical learning outcomes** |
| --- | --- | --- | --- | --- | --- |
| **Key competencies** | | | | | |
| **Principles of evidence-based health care** | Terminology and understanding | 3 | Research Methodology 312 | Comprehension | Understand and apply the concept of evidence-based nutrition |
| **Formulating questions** | Identify knowledge gaps | - | - | - | - |
|  | Using PICO format | - | - | - | - |
|  | Identify various types of questions | - | - | - | - |
| **Literature search strategy** | Identify best study design for a specific type of question | 3 | Research Methodology 312 | Application, Skill | Select an appropriate study design according to the research problem |
|  | Design a relevant search strategy | 3 | Research Methodology 312 | Application, Skill | Plan a literature search using a systematic approach |
|  | Identify appropriate databases | - | - | - | - |
|  | Performing an electronic search | 2,3 | Applied Food Science 254, Research Methodology 312 | Skill | Perform a literature search using a systematic approach |
| **Critical appraisal** | Appraise systematic reviews | - | - | - | - |
|  | Appraise randomised controlled trials | - | - | - | - |
|  | Appraise cohort studies | - | - | - | - |
|  | Appraise case-control studies | - | - | - | - |
|  | Appraise cross-sectional studies | - | - | - | - |
|  | Appraise diagnostic studies | - | - | - | - |
|  | Appraise qualitative studies | - | - | - | - |
|  | Interpret research findings | - | - | - | - |
|  | Translate outcomes into summary statistics | - | - | - | - |
|  | Appraisal not linked to specific study design | 1,3 | Nutrition 142, Biostatistics and Epidemiology 322, Research Methodology 312 | Comprehension, Application, Evaluation | Read medical literature critically and make sure that you understand potential errors/mistakes and ambiguousness in published medical articles as well as the results at which the authors arrive so that you apply them with confidence |
| **Applying the evidence** | Considering application of literature | - | - | - | - |
| **Enabling competencies** | | | | | |
| **Biostatistics** | Hypothesis testing | 1,3 | Nutrition 142, Biostatistics and Epidemiology 322 | Comprehension | Describe the basic process to prove or refute hypothesis and in doing so create theories |
|  | Sampling | 1,3 | Health in Context 111, Research Methodology 312 | Comprehension, Application | Describe, identify and apply random and non-random methods of sampling |
|  | Descriptive statistics | 1,3 | Health in Context 111, Biostatistics and Epidemiology 322 | Knowledge, Comprehension, Application | Descriptive statistics, probability, hypothesis testing, parametric and non-parametric methods, regression and correlation analysis, analysis of variance with special reference to application in dietetics |
|  | Probability | 1,3 | Health in Context 111,  Biostatistics and Epidemiology 322 | Knowledge, Comprehension, Application, Skill |  |
|  | Estimation and uncertainty | 3,4 | Biostatistics and Epidemiology 322, Research Methodology 413 | Comprehension |  |
| **Epidemiology: quantitative research aspects** | Study design | 1,3 | Health in Context 111, Nutrition 142, Research Methodology 312 | Knowledge, Comprehension | Name and define the types of research used in human research |
|  | Reliability and validity | 2,3,4 | Applied Food Science 254, Practical Training 272, Practical Training 374, Research Methodology 312, Therapeutic Nutrition 478 | Comprehension, Application, Skill | Motivate the reliability of the information gathered according to the methodology |
|  | Measures of health and disease occurrence | 1,2,3 | Health in Context 111, Therapeutic Nutrition 244, Community Nutrition 244, Research Methodology 312, Therapeutic Nutrition 378, Biostatistics and Epidemiology 322 | Knowledge, Comprehension, Skill | Describe the difference between and uses of the following indicators: rates, incidence, prevalence, mortality and morbidity |
|  | Association, causation, and effect | 1,3 | Health in Context 111, Research Methodology 312 | Knowledge, Comprehension, Skill | Define and differentiate between causes and risk factors |
|  | Ethical considerations | 3,4 | Research Methodology 312, Community Nutrition 478 | Knowledge, Comprehension | Identify and discuss the ethical principles applicable to research |
|  | Diagnostic principles | 1,3 | Health in Context 111, Research Methodology 312, Biostatistics and Epidemiology 322 | Knowledge, Skill | Define the terms sensitivity and specificity |
|  | Screening principles | - | - | - | - |
|  | Bias | 3 | Research Methodology 312 | Knowledge, Comprehension | Discuss and identify the following biases: confounding bias, selection bias, information bias |
|  | Survey methods | 3 | Research Methodology 312 | Comprehension, Application | Plan and compile a questionnaire using a systematic approach |
| **Qualitative research aspects** | Interviews, focus groups and observations | 3 | Research Methodology 312 | Comprehension, Application | Describe and apply the different methods of data gathering, interviews, focus groups and observations |
|  | General use of qualitative research | 3 | Research Methodology 312 | Knowledge | Discuss advantages and disadvantages of qualitative research |
| **Basics skills of searching electronic databases** | Identify appropriate search terms | - | - | - | - |
|  | Use of Medical Subject Headings (MeSH) in search | - | - | - | - |
|  | Explode MeSH terms | - | - | - | - |
|  | Use appropriate limits/filters | - | - | - | - |
|  | Use of Boolean operators | - | - | - | - |
|  | Combine search terms in final search strategy | - | - | - | - |
| **Philosophy of critical enquiry** | Critical thinking and reflection | 1,2,3 | Nutritional Status Assessment 144, Foods 144, Nutrition 142, Applied Food Science 254, Food Production and Systems 214, Community Nutrition 244, Biostatistics and Epidemiology 322 | Application, Analysis, Evaluation | Collect information, analyse it, evaluate it critically and organise it |
|  | Problem-solving | 2,4 | Community Nutrition 244, Community Nutrition 478 | Knowledge, Application | Identify and solve problems according to responsible, critical and creative thinking |
| **Communication** | Presentation skills | 1,2,4 | Foods 144, Nutrition 142, Applied Food Science 254, Food Production and Systems 214, Therapeutic Nutrition 244, Community Nutrition 244, Food Service Management 476, Community Nutrition 478 | Skill | Communicate effectively by using and applying verbal and visual methods |
|  | Patient communication | 2,3,4 | Community Nutrition 244, Psychology for Health Sciences 242, Community Nutrition 244, Nutrition in the Life Cycle 214, Managerial Principals 377, Therapeutic Nutrition 478 | Knowledge, Comprehension, Application, Skill | The student must apply her communication and education techniques during the diet consultation of this patient before discharge |

**Evidence-based health care competencies in the undergraduate physiotherapy programme**

| **Competencies** | **Content covered** | **Year** | **Module** | **Type of learning outcome** | **Quotes of typical learning outcomes** |
| --- | --- | --- | --- | --- | --- |
| **Key competencies** | | | | | |
| **Principles of evidence-based health care** | Terminology and understanding | 3,4 | Research Methods 372, Physiotherapy practice 474, Research Methods 472 | Comprehension | Understand the principles of evidence-based practice |
| **Formulating questions** | Identify knowledge gaps | - | - | - | - |
|  | Using PICO format | 3 | Research Methods 372 | Application | Be able to design a secondary research question while using the PICO method |
|  | Identify various types of questions | - | - | - | - |
| **Literature search strategy** | Identify best study design for a specific type of question | - | - | - | - |
|  | Design a relevant search strategy | - | - | - | - |
|  | Identify appropriate databases | - | - | - | - |
|  | Performing an electronic search | 3,4 | Applied Physiotherapy 373, Research Methods 372, Applied Physiotherapy 473 | Skill | Be able to search effectively for published physiotherapy research articles using the most common medical databases |
| **Critical appraisal** | Appraise systematic reviews | - | - | - | - |
|  | Appraise randomised controlled trials | - | - | - | - |
|  | Appraise cohort studies | - | - | - | - |
|  | Appraise case-control studies | - | - | - | - |
|  | Appraise cross-sectional studies | - | - | - | - |
|  | Appraise diagnostic studies | - | - | - | - |
|  | Appraise qualitative studies | - | - | - | - |
|  | Interpret research findings | - | - | - | - |
|  | Translate outcomes into summary statistics (e.g. NNT or RRR) | - | - | - | - |
|  | Appraisal not linked to specific study design | 3,4 | Applied Physiotherapy 373, Applied Physiotherapy 473, Clinical Physiotherapy 374 , Clinical Physiotherapy 474, Research Methods 372, Research Methods 472 | Application, Analysis | Be able to evaluate literature using physiotherapy-related critically appraisal tools |
| **Applying the evidence** | Considering application of literature | - | - | - | - |
| **Enabling competencies** | | | | | |
| **Biostatistics** | Hypothesis testing | - | - | - | - |
|  | Sampling | 1 | Health in Context 111 | Comprehension | Describe the different random sampling techniques (in your own words) and identify examples thereof |
|  | Descriptive statistics | 3 | Research Methods 372 | Comprehension | Understand basic research statistical concepts |
|  | Probability | 1 | Health in Context 111 | Knowledge, Comprehension | Apply the basic rules of probability on frequencies of 2×2 tables |
|  | Estimation and uncertainty | - | - | - | - |
|  | Meta-analysis | 4 | Research Methods 472 | Application | Apply basis statistical concepts on primary research data (meta-analysis) |
| **Epidemiology: quantitative research aspects** | Study design | 1,3 | Health in Context 111,  Research Methods 372 | Knowledge, Comprehension | Describe relevant study designs that are relevant to physiotherapy research |
|  | Reliability and validity | - | - | - | - |
|  | Measures of health and disease occurrence | 1,3 | Health in Context 111, Pathology 312 | Knowledge, Comprehension | Give the meaning of incidence and prevalence in your own words |
|  | Association, causation, and effect | 1 | Health in Context 111 | Knowledge, Skill | Calculate and interpret the components of a relative risk (EER, CER, ARR, NNT, RRR); you must memorise these formulae |
|  | Ethical considerations | - | - | - | - |
|  | Diagnostic principles | 1 | Health in Context 111 | Knowledge, Skill | Calculate the sensitivity and specificity of procedures in medical examples, and give an interpretation thereof |
|  | Screening principles | - | - | - | - |
|  | Bias | - | - | - | - |
|  | Survey methods | - | - | - | - |
| **Qualitative research aspect** | Interviews, focus groups and observations | - | - | - | - |
|  | General use of qualitative research | - | - | - | - |
| **Basics skills of searching electronic databases** | Identify appropriate search terms | - | - | - | - |
|  | Use of Medical Subject Headings (MeSH) in search | - | - | - | - |
|  | Explode MeSH terms | - | - | - | - |
|  | Use appropriate limits/filters | - | - | - | - |
|  | Use of Boolean operators | - | - | - | - |
|  | Combine search terms in final search strategy | - | - | - | - |
| **Philosophy of critical enquiry** | Critical thinking and reflection | 1,3,4 | Personal and Professional Development 111, Applied Physiotherapy 373, Applied Physiotherapy 473 | Attitude | These tasks stimulate curiosity in the students, encouraging them to actively explore and seek out new evidence. The student is responsible for analysing and presenting evidence in appropriate ways and in support of their own response to the problem. |
|  | Problem-solving | 3,4 | Applied Physiotherapy 373, Applied Physiotherapy 473 | Application | The emphasis of Applied Physiotherapy 3 is on integrating theory and practice, problem-solving and clinical reasoning |
| **Communication** | Presentation skills | 3,4 | Applied Physiotherapy 373, Applied Physiotherapy 473, Research Methods 472 | Skill | Be able to deliver a scientific oral report of the research outcomes of your research group by making use of PowerPoint |
|  | Patient communication | 3,4 | Clinical Physiotherapy 374, Clinical Physiotherapy 474 | Skill | Communicate accurately with …, clients, carers … |

**Evidence-based health care competencies in the undergraduate speech, language and hearing therapy programme**

| **Competencies** | **Content covered** | **Year** | **Module** | **Type of learning outcome** | **Quotes of typical learning outcomes** |
| --- | --- | --- | --- | --- | --- |
| **Key competencies** | | | | | |
| **Principles of evidence-based health care** | Terminology and understanding | - | - | - | - |
| **Formulating questions** | Identify knowledge gaps | 3 | Psychology 318 | Comprehension | To encourage students to think about important research issues affecting South Africa |
|  | Formulating questions using PICO format | - | - | - | - |
|  | Formulating questions | 3,4 | Psychology 318, Speech Pathology 364, Speech Pathology 472 | Application | Formulate a research question in relation to the subject field |
|  | Identify various types of questions | 4 | Speech Pathology 472 | Application | Select a research approach and a design suitable for answering the research question |
| **Literature search strategy** | Identify best study design for a specific type of question | - | - | - | - |
|  | Design a relevant search strategy | - | - | - | - |
|  | Identify appropriate databases | - | - | - | - |
|  | Performing an electronic search | 1,3 | Speech Pathology 162, Speech Pathology 364 | Skill | Conduct a literature search |
| **Critical appraisal** | Appraise systematic reviews | - | - | - | - |
|  | Appraise randomised controlled trials | - | - | - | - |
|  | Appraise cohort studies | - | - | - | - |
|  | Appraise case-control studies | - | - | - | - |
|  | Appraise cross-sectional studies | - | - | - | - |
|  | Appraise diagnostic studies | - | - | - | - |
|  | Appraise qualitative studies | - | - | - | - |
|  | Interpret research findings | - | - | - | - |
|  | Translate outcomes into summary statistics | - | - | - | - |
|  | Appraisal not linked to specific study design | 3 | Psychology 318 | Skill | To develop critical skills in evaluating psychological research |
| **Applying the evidence** | Considering application of relevant literature | - | - | - | - |
| **Enabling competencies** | | | | | |
| **Biostatistics** | Hypothesis testing | - | - | - | - |
|  | Sampling | 3 | Psychology 318 | Comprehension | To understand the logic of quantitative research methods, including sampling … |
|  | Basic and descriptive statistics | 3 | Psychology 318 | Comprehension | To introduce students to basic concepts in data analysis and develop their skills to do basic statistical analysis |
|  | Probability | - | - | - | - |
|  | Estimation and uncertainty | - | - | - | - |
|  | Meta-analysis | - | - | - | - |
| **Epidemiology: quantitative research aspects** | Study design | 3 | Psychology 318, Speech Pathology 364 | Comprehension | Understand the difference between descriptive and intervention studies |
|  | Reliability and validity | - | - | - | - |
|  | Measures of health and disease occurrence | 3,4 | Speech Pathology 378, Speech Pathology 413 | Knowledge, Comprehension | Have basic knowledge of the different aetiologies and prevalence of neurogenic communication disorders |
|  | Association, causation, and effect | 3 | Psychology 318 | Comprehension | To understand the logic of quantitative research methods, including … causal claims … |
|  | Ethical considerations | 3 | Speech Pathology 364, Speech Pathology 472 | Comprehension | Explain the importance of research ethics |
|  | Diagnostic principles | 1,2 | Speech Pathology 162, Clinical Speech Pathology 274 | Application, Analysis | Be able to determine test performance characteristics |
|  | Screening principles | 1 | Speech Pathology 162 | Comprehension | To explain the epidemiological concepts involved in screening or early detection |
|  | Bias | - | - | - | - |
|  | Survey methods | - | - | - | - |
| **Qualitative research aspects** | Interviews, focus groups and observations | - | - | - | - |
|  | General use of qualitative research | 3 | Psychology 318 | Comprehension, Analysis | To differentiate between the qualitative research approaches that have been covered in this course |
| **Basics skills of searching electronic databases** | Identify appropriate search terms | - | - | - | - |
|  | Use of Medical Subject Headings (MeSH) in search | - | - | - | - |
|  | Explode MeSH terms | - | - | - | - |
|  | Use appropriate limits/filters | - | - | - | - |
|  | Use of Boolean operators | - | - | - | - |
|  | Combine search terms in final search strategy | - | - | - | - |
| **Philosophy of critical enquiry** | Critical thinking and reflection | 3,4 | Psychology 318, Speech Pathology 411, Clinical Speech Pathology 474 | Comprehension, Skill | Have the ability to use evaluation procedures/materials appropriately, analytically and with critical consideration |
|  | Problem-solving | - | - | - | - |
| **Communication** | Presentation skills | - | - | - | - |
|  | Patient communication | 2 | Clinical Speech Pathology 274 | Skill | Communication of results and interpretation of results with parents/guardians in an appropriate manner |
